# Bridging Social Capital and Individual Earnings: Evidence for an Inverted U

**DOI:** 10.1007/s11205-015-0980-z

**Published:** 2015-05-23

**Authors:** Katarzyna Growiec, Jakub Growiec

**Affiliations:** Department of Psychology of Personality, University of Social Sciences and Humanities, ul. Chodakowska 19/31, 03-815 Warsaw, Poland; Department of Quantitative Economics, Warsaw School of Economics, al. Niepodległości 162, 02-554 Warsaw, Poland; Narodowy Bank Polski, ul. Świętokrzyska 11/21, 00-919 Warsaw, Poland

**Keywords:** Bridging social capital, Social trust, Earnings, Nonlinearity

## Abstract

Based on data on a cross section of individuals surveyed in the 1999–2002 wave of World and European Values Surveys, we investigate the multilateral associations between bridging social capital, individuals’ earnings, as well as social trust and employment status. Our analysis provides robust evidence that the relationship between bridging social capital and earnings is inverted-U shaped. We carry out a range of tests in order to ascertain that this result is not driven by regressor endogeneity or omitted variables bias. We also identify significant interaction effects between bridging social capital, social trust, and employment status.

## Introduction

Social capital, defined on the basis of density and diversity of individuals’ social networks (Lin 2001), is often argued to be an important determinant of their economic and psychological well-being. For example, it has been found that inhabitants of richer countries are relatively more willing to form and maintain numerous social ties with people dissimilar to themselves, whereas in less affluent areas, people are more likely to restrict their social ties to narrow groups consisting primarily of family members. Similar associations have also been found within societies, prompting the hypothesis that various forms of social capital can have important consequences for individuals’ well-being.

In particular, as confirmed in numerous empirical studies, social capital has sizeable effects for individuals’ earnings. Under the network operationalization of social capital (Lin 2001; Sobel 2002; Kadushin 2012) and following Putnam’s (2000) distinction between bridging social capital (social ties with dissimilar others) and bonding social capital (social ties with similar others), these two types of social capital have been found to have opposing influences on earnings. But for several exceptions, it is usually argued that bridging social capital increases earnings whereas bonding social capital decreases them. Results of these studies are reviewed in Table 1.^1^

In this paper, we revisit these earlier empirical findings on the basis of worldwide World and European Values Surveys (WVS and EVS) data. Our claim is that the literature has overlooked an important possibility of a nonlinear association between bridging social capital and individuals’ earnings. This is a potentially important gap in the literature because there are valid reasons to seek an inverted-U shaped relationship between bridging social capital and earnings: on the one hand, bridging social ties can be a source of valuable resources, increasing one’s earning potential (cf. e.g., Podolny and Baron 1997; Burt 2005); on the other hand, maintaining social ties necessarily incurs time costs and thereby at the margin, limits the potential for additional earnings. Hence, if the individual’s investment in social ties is already sufficiently high (“too high”), these time costs can outweigh the pecuniary benefits of accumulating additional bridging social capital.^2^

The current study provides strong empirical support for the aforementioned theoretical hypothesis. Based on data on a cross section of individuals surveyed in the 1999–2002 wave of World and European Values Surveys, we identify the curvature and strength of relationship between bridging social capital and individuals’ earnings, controlling for respondents’ social trust, employment status, education, and a wide range of other social characteristics. We find that the association between bridging social capital and earnings indeed has an inverted-U shape. Simultaneously, we also confirm that social trust is positively related to individuals’ earnings.

The methodological strength of our empirical analysis lies with the fact that in addition to looking for nonlinear effects, we also identify the consequences of the potential presence of omitted variables and the endogeneity of social capital in earnings regressions. We tackle the problem of social capital endogeneity by applying the instrumental variables (IV) estimation technique as well as testing the validity and identification properties of instrumental variables used in each regression specification.^3^ We also identify an important role of several confounding variables, able to influence both sides of the estimated equation. We conclude that omitting these variables and disregarding the endogeneity problem could have biased some of the earlier results, but it does not affect the main empirical finding of the current study: that the association between bridging social capital and individuals’ earnings has the inverted-U shape.

The remainder of the paper is structured as follows. Section 2 discusses the relevant theoretical literature underlying our empirical investigation. Section 3 discusses empirical measurement and instrumentation issues. Our main results are presented in Section 4. Section 5 discusses a range of robustness checks. Section 6 concludes.

**Table 1:** Summary of results of selected empirical studies

Study	Data	Operationalization of social capital	Dependent variable	Estimation method	Impact on dependent variable
Beugelsdijk and Smulders (2003)	54 European regions 1990 (EVS)	Bonding: importance of family, friends, and acquaintances. Bridging: membership in certain voluntary organizations	Regional economic growth 1950–1998	2SLS [endogenous bridging]	Bonding: weak−. Bridging: +
Mouw (2003)	USA panel 1982–1994 (NLSY)	Present job found though social contacts	Log wage	OLS / FE	Contacts: 0
Franzen and Hangartner (2006)^ a^	15 countries, 2011 (ISSP)	Bonding: present job found through contacts with kin or close friends. Bridging: present job found through acquaintances. Controlling for the number of friends	Log wage	OLS / ML	Bonding: −. Bridging: −
Kim (2009)	Chicago lawyers 1995 (dedicated study)	Network characteristics: Network constraint, Network bridge, Strong ties	Log income	OLS	Strong ties: −. Network constraint: 0. Network bridge: 0
Sabatini (2009)	Italy 1998–2002 (household survey)	Bonding: strength of family ties. Bridging: informal networks of friends and neighbors	Log income per person in household	Structural equations model [endogenous bridging]	Bonding: −. Bridging: −
Alesina and Giuliano (2010)^ b^	78 countries, mostly 1999–2001 (WVS)	Bonding: strength of family ties and the tendency to form kinship groups based on unconditional loyalty	Home production (from time use survey)	OLS	Bonding: +
Growiec and Growiec (2010)	Poland 2005 (Social Diagnosis)	Bonding: number of often contacted family members. Bridging: number of often contacted friends	Log income per person in household	OLS	Bonding: 0. Bridging: +
Zhang et al. (2011)	USA panel 1987/1994 (NSFH)	Bonding: social activity and help, kin and friends. Bridging: participation in organizations	Log income	OLS	Bonding: 0. Bridging: +

*OLS* Ordinary Least Squares, *2SLS* Two-Stage Least Squares, *FE*Fixed Effects, *ML* Maximum Likelihood, *EVS*European Values Survey, *NLSY* National Longitudinal Study of Youth, *ISSP* International Social Survey Program, *WVS* World Values Survey, *NSFH* National Survey of Families and Households

^a^In Franzen and Hangartner (2006), bridging social capital is measured according to a qualitatively distinct operationalization than ours

^b^In Alesina and Giuliano (2010), the original dependent variable is home production, a proxy variable negatively related to income per person in the household

## Related Literature

Regarding the underlying definition of social capital, we build on the principal idea to operationalize bridging and bonding social capital via the characteristics of individuals’ social networks (cf. Lin 2001). Such an approach is especially fruitful analytically because it enables one to delineate people’s objective behavior (maintaining social contacts with others) from social norms (trust, reciprocity). Indeed, definitions which fail to acknowledge this distinction have been forcefully criticized in the literature, with the implication that they may even render the entire social capital concept defective (Bjørnskov and Sønderskov 2013).

The social network perspective on social capital is widely shared in the literature (Lin 2001; Kadushin 2002; Li et al. 2005; Burt 2005); moreover, this position leads to being more specific on social networks people form and, as a consequence, to what resources they have access (Bourdieu 1986; Gittell and Vidal 1998; Lin 2001).^4^ Putnam’s (2000) distinction between bridging social capital (social ties with dissimilar others) and bonding social capital (social ties with similar others) has by now become a standard in social capital studies; on the other hand, there is still little congruence in the literature on the appropriate empirical method of social capital measurement. In micro-level analyses, bridging social capital is often measured as the frequency of social contact with people in a different social-economic position to oneself. In large-scale survey datasets such as the WVS and EVS used here, however, there always remains the problem of data availability. In the current paper, this problem forced us to omit bonding social capital from the study and focus on bridging social capital only.^5^

Our main empirical hypothesis, on the other hand, is rooted in the theoretical literature which deals with the impact of social capital and trust on economic performance at the level of individuals, communities, regions, and whole countries. Some sociologists argue that bridging social capital, as opposed to bonding social capital, goes together with civil liberties and the support for gender and racial equality, and strengthens the functioning of democracy by reducing corruption (Putnam et al. 1993; Putnam 2000). On the other hand, “bonding social capital (as distinct from bridging social capital) has negative effects for society as a whole, but may have positive effects for the members belonging to this closed social group or network” (Beugelsdijk and Smulders 2003). Beugelsdijk and Smulders (2003) proceed to show that bridging social capital is empirically good for economic growth at the level of European regions, whereas bonding social capital is bad for growth.

Bridging social capital has also been found to be individually beneficial for those who possess it, though. Granovetter’s (1973) prominent discovery is that weak ties (i.e., ties between dissimilar people) are more useful for finding better jobs than strong ties (between similar people). Friendship ties have also been shown to be positively related to individuals’ wages and upward mobility in the workplace (Podolny and Baron 1997; Słomczyński and Tomescu-Dubrow 2005). Most strongly perhaps, Burt (2005) claims that bridging social capital, as opposed to bonding social capital, is positively related to individuals’ economic performance, creativity, social trust, and happiness. The question whether diversified social networks indeed improve the individuals’ earnings potential remains unsettled, though: recent research from Franzen and Hangartner (2006) indicates that using social networks might not necessarily increase the monetary payoff but improve the nonpecuniary characteristics of the job like better career perspectives instead. Another caveat is that social networks are endogenous to individuals’ economic position because, keeping the number of hours worked constant, high-paying jobs tend to generate relatively more opportunities for building resourceful bridging ties with dissimilar others than low-paying jobs.

Finally, in two closely related papers (Growiec and Growiec 2010, 2014b), we have put forward theoretical models which formalize the hypothesis that bridging social capital can have a nonlinear, inverted-U shaped impact on individuals’ earnings. The hypothesized nonlinearity comes up as a result of the interplay of two opposing forces: (1) the positive productivity spillover stemming from bridging social capital in the production function, and (2) the time cost associated with the accumulation and maintenance of social capital by individuals.^6^ To our knowledge, this hypothesis has not been addressed in the empirical literature so far—which is unfortunate because a nonlinearity could potentially explain some of the discrepancies among the empirical results obtained so far.

Moreover, in the latter contribution we argue that bridging social capital and social trust can also form virtuous and vicious circles, leading to multiple equilibria in economic performance. In Section 5.5, we test these predictions empirically by checking the signs and statistical significance of interaction terms between both bridging social capital, trust, and employment status, leading to mixed results.

An extended review of the background literature can also be found in our two related publications (Growiec and Growiec 2014a, b).

## Measurement and Instrumentation

The current study is based on data from the World and European Values Surveys (WVS and EVS) which are an international survey program based on a standardized questionnaire. The survey is conducted in each member country by a local public opinion survey institution, in the local language, on a representative sample of the country’s population aged 18+. We use cross-sectional data from the 1999–2002 wave of the WVS and EVS, which was the only wave to include an extended list of questions relevant to the measurement of bridging social capital. For our main regressions, we use around 20–22 thousand observations from 31 countries.^7^

### Measurement of Bridging Social Capital and Social Trust

Bridging social capital refers to forming social ties across social cleavages, outside of the family and close neighborhoods, requiring people to transcend their simple social identity (Gittell and Vidal 1998; Putnam 2000; Leonard 2008). It should be identified by people’s objective behavior and not the social norms to which they adhere. In line with this definition, we measure it as a summary scale based on the following questions: “How often do you spend time with your friends”, “How often do you spend time socially with your colleagues from work or your profession”, “How often do you spend time socially with people at sports clubs, voluntary or service organizations”, with available answers: weekly, once or twice a month, only a few times in a year, not at all. This summary scale has Cronbach’s alpha of about 0.52 (depending on the sample) and its choice is optimal in the sense that its validity cannot be improved by removing any of its constituent items. Our approach is also justified by the fact that factor analysis confirms that the scale is unidimensional, and all items are included in the primary factor with similar loadings.^8^

We simultaneously monitor individuals’ self-reported social trust, measured by their choice among two statements: “Most people can be trusted” or “One can’t be too careful [with other people]”. We shall also distinguish between individuals’ own level of trust towards strangers and the degree to which they themselves are trusted. As a proxy measure of the latter, we shall use the average level of trust in the individuals’ reference group. To this end, we stratify individuals by their country of residence and education level.

### Measurement of Earnings

Our dependent variable is constructed on the basis of the WVS ten-degree scale of *total household income*. The exact survey question is: “Here is a scale of incomes. We would like to know in what group your household is, counting all wages, salaries, pensions, and other incomes that come in. Just give the letter of the group your household falls into, before taxes and other deductions.” Respondents are asked to identify the earnings decile to which their household belongs.

To bring this measure as close as possible to a measure of *individuals’ earnings*, we transform the aforementioned WVS scale into a scale of equivalent household earnings. To this end, we divide total household income by the square root of the total number of persons in the household, including children.^9^ As the explained variable in our preferred regression equation, we take the natural logarithm of such a measure (denoted as *income_ln_eq*). This approach allows us to treat our model as a Mincerian earnings equation (Mincer 1974). Indeed, the associated literature (see Heckman et al. 2003, for a review) provides a range of theoretical and empirical arguments that the estimated relationship between individuals’ years of schooling, age, age squared, as well as other socio-economic characteristics, and earnings, should take the *log*-linear form.

As a robustness check, we confirm that our main findings are robust to replacing this variable with the original WVS measure of household incomes, its logarithm, as well as household income per person in the household. They also remain in force when the model is estimated as an ordered logit, i.e., when the explained variable is treated as an ordinal and not an interval scale.

**Fig. 1:**
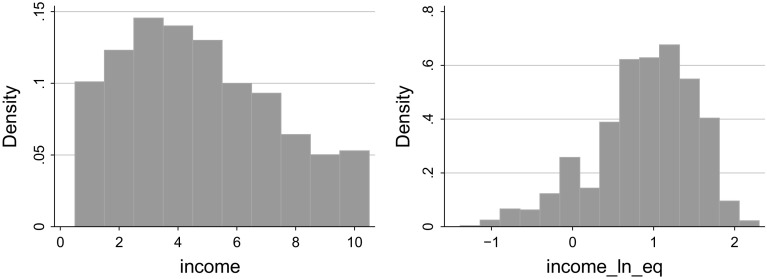
Histograms: household incomes (*left panel*) and log equivalent incomes (*right panel*)

Histograms presented in Fig. 1 reveal that the distribution of household earnings tend to be right-skewed despite the fact that respondents were supposed to divide themselves in deciles. This can be a consequence of cognitive bias of respondents in the sample (e.g., due to comparing one’s wage to the mean instead of the median wage in the population) or a systematically wider incidence of missing data from households located in upper deciles of the income distribution. Upon applying the logarithmic transformation, we obtain a left-skewed distribution, though. This property is shared also by log equivalent earnings, our dependent variable.

Since our explained measure is naturally sensitive to the number of adult persons in the household, we control for household size (*hh_size*: number of persons aged 18+ in the household) in all our regressions. Given that the WVS definition refers to income deciles within the respondent’s country, we always include country fixed effects.

### Instrumental Variables

If there is regressor endogeneity, i.e., if apart from the sought causal link from individuals’ bridging social capital stocks to their earnings there also exists a reverse causal link from earnings to social ties, the strength of the actual impact of bridging social capital can only be identified thanks to an auxiliary use of instrumental variables. Such variables, by construction, should be exogenous, i.e., uncorrelated with the error term of the estimated regression equation, and sufficiently strongly correlated with the endogenous regressors.

This said, one has to admit that it is a very hard task to find good (i.e., both exogenous and strong) instruments for bridging social capital in cross-sectional survey data. Altough the results of statistical tests which we have carried out are positive, from the theoretical perspective we cannot fully exclude that some of these variables are nevertheless endogenous.

Our final list of instruments includes a measure of interest in politics (survey question: “Is politics important in your life?”) and membership in organizations related to education, arts, music or cultural activities. Apart from their favorable statistical properties in our dataset, these variables have been identified as related to bridging social capital and *not* directly related to income in several notable contributions.

- *Interest in politics* Interest in politics makes people more engaged in the local community and better connected to others, thus increasing their social capital (Halpern 2005). Engagement in political campaigns and social activism (e.g., boycotting, signing petitions, donating money, contacting officials) are among the important ways of being socially active. Research shows that income and interest in politics (or voter turnout) are not directly related, however (Hill and Leighley 1992; Barnes 2013)—other factors like social class (Hill and Leighley 1992) or unemployment and unionization levels (Barnes 2013) are relevant here.
- *Membership in organizations* According to Putnam (2000) and Halpern (2005), associational participation is positively related to individuals’ social capital resources. In particular, it “emancipates individuals to trust others beyond close personal relationships” (Park and Subramanian 2012) and thus relates to their bridging social capital. Although participation in voluntary groups is generally more prevalent among individuals with higher status and prestige, its relationship with individual incomes is more nuanced (Bekkers et al. 2012). In particular, membership in organizations related to education, arts, music or cultural activities belongs to the category of *expressive* membership (Weber 1978), which—as opposed to instrumental membership (in political parties, labor unions, interests groups)—is unrelated to individual incomes (Bekkers et al. 2012).

The IV procedure has been carried out in the following way.^10^ We begin with estimating the first-stage regression equation where the endogenous regressor (bridging social capital) is taken as the dependent variable, and the aforementioned set of instruments as well as the exogenous regressors included also in the main regression equation—are taken as independent variables. The first-stage equation is estimated with OLS. The theoretical (predicted) values from this model are stored for later use in the second stage. In the second stage, we set up the main regression equation explaining earnings, including these theoretical values instead of the endogenous regressors alongside the exogenous regressors. Our first-stage regression results (not reported) indicate that, in line with the associated literature (Halpern 2005; Kadushin 2012), bridging social capital is positively determined by interest in politics, membership in organizations related to education, arts, music or cultural activities, social trust (see also Miyata et al. 2012; Ishiguro and Okamoto 2013), employment status, education, freedom of choice and control, and negatively affected by housewife status and (puzzlingly) membership in sports and recreation clubs. It also follows an inverted-U shaped relationship with individuals’ age.

The results of Sargan tests indicate that our instruments are valid, whereas underidentification tests prove that our auxiliary regressions are able to identify the endogenous regressors correctly with instruments. Anderson–Rubin tests indicate that the endogenous variable is statistically significant in the main equation. At the same time, Chi-square endogeneity tests confirm that bridging social capital is indeed correlated with the error term of the OLS regression, and thus OLS results are biased because of regressor endogeneity.

## Results

Let us now proceed to the presentation of our principal results. We shall first provide a graphical demonstration of the inverted-U relationship between bridging social capital and individual earnings. In the second subsection, we will provide our main set of empirical results which confirms robustness of this result to a range of control variables and social capital endogeneity. Further extensions and robustness checks are relegated to the consecutive section.

### Bridging Social Capital Versus Earnings: Empirical Evidence for an Inverted U

**Fig. 2:**
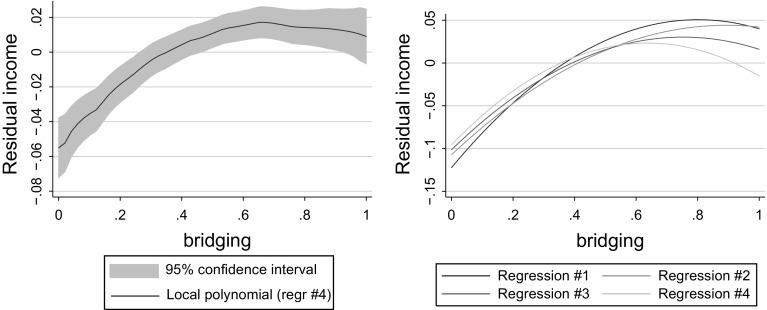
The relationship between bridging social capital and residual incomes. Evidence for an inverted U

To visualize the nonlinear association between bridging social capital and individual earnings, we have carried out a simple two-step procedure. In the first step, we have regressed a range of explanatory variables—all variables from regression (4) in Table 2 excluding bridging social capital—on individual earnings and stored the residuals. In the second step, we have performed a kernel-weighted local polynomial regression (with the Epanechnikov kernel),^11^ explaining these residual earnings with bridging social capital. The results are presented in the left panel of Fig. 2. Using non-parametric kernel smoothing techniques allows us to remain agnostic about the functional form of the relationship between both variables and let the data decide about it instead.

We find that the data strongly favor a non-monotonic, inverted-U shape of the relationship between bridging social capital and (residual) earnings. In fact, this regression can also be very well fitted with a very parsimonious quadratic specification.

Furthermore, as demonstrated in the right panel of Fig. 2, the shape of the bridging social capital–earnings relationship remains essentially unaffected also when one limits the number of control variables in the regression. The respective parabolas represent quadratic regressions of bridging social capital on residual earnings, with residuals computed taking explanatory variables from regressions (1–4) in Table 2, respectively. However, when one fails to control for the relevant regressors, the maximum appears shifted to the right and the range of positive impact appears wider.^12^

This principal result can be viewed as empirical support for Proposition 4 in Growiec and Growiec (2010) and Proposition 5 in Growiec and Growiec (2014b),^13^ originating from theoretical models. On the other hand, it stands in sharp contrast to earlier empirical results due to, e.g., Beugelsdijk and Smulders (2003), Florida (2004) and Growiec and Growiec (2010), who have only sought for a linear relationship, and found a positive one.

The appearance of a range of negative dependence (or under a conservative interpretation, lack of dependence) between individuals’ earnings and their bridging social capital stocks may be due to two reasons. First, from the psychological perspective, the amount of time people spend with their friends, co-workers, people from one’s church or voluntary organization, etc., can be heavily dependent on their choice between materialist and post-materialist values (Inglehart and Baker 2000). Respondents who have an inclination towards the latter may be relatively more willing to devote additional time to their circle of friends and acquaintances and withdraw some of their activity from the labor market, thus lowering their earnings. The second mechanism is an economic one and applies to employed people who work long hours. Given their tight time constraints, the positive external effects of additional bridging social capital on their earnings will be likely dominated by time-substitution effects.

### Bridging Social Capital and Social Trust Versus Individual Earnings

The key regression results of the current study are contained in Table 2. Models (1–4) have been estimated with OLS. By gradually increasing the number of relevant control variables from one equation to another, we quantify the extent to which the initial estimates have been affected by omitted variables bias.^14^ Models (5–8), on the other hand, have been estimated with instrumental variables (IV), allowing us to control for endogeneity of bridging social capital in earnings regressions. Indeed, our Chi-square endogeneity tests confirm that bridging social capital is correlated with the error term in OLS regressions, and thus one should expect OLS results to be biased.

As shown in Table 2, the result of an inverted-U shaped association between bridging social capital and earnings is robust to controlling for social capital endogeneity and confounding variables. Statistical significance at 1 % level is maintained for all regression specifications, and the difference between the estimated coefficients on linear and quadratic bridging social capital terms declines as we move from regression (1) towards (8). The maximum of the parabola is shifted from 0.77 in regression (1) to 0.71 in (4) and just 0.57 in (8), fully corroborating the results presented graphically in Fig. 2.

Our another finding is that earnings are also positively related to social trust, even when the (endogenous) bridging social capital stock is controlled for. This result refers both to the extent to which one trust others and the level to which she experiences trust in return (cf. Knack and Keefer 1997; Zak and Knack 2001). On average, and keeping other things equal, the more individual trusts others, the better is she off. It supports the idea that social trust enhances incomes by opening individuals to more beneficial situations (Growiec 2011). High trust standards also make contacts at the workplace more favorable in terms of information flow, less stressful, and thus they effectively reduce transaction costs in doing business (Ostrom and Walker 2003; Williamson 1981).

**Table 2:** Bridging and bonding social capital as determinants of individual earnings: a comparison of regression specifications

Variables	(1)	(2)	(3)	(4)	(5)	(6)	(7)	(8)
	inc_ln_eq	inc_ln_eq	inc_ln_eq	inc_ln_eq	inc_ln_eq	inc_ln_eq	inc_ln_eq	inc_ln_eq
	OLS	OLS	OLS	OLS	IV	IV	IV	IV
Bridging	0.456*** [8.195]	0.484*** [8.938]	0.434*** [7.776]	0.420*** [7.642]	12.21*** [5.194]	6.706*** [4.553]	5.910*** [3.463]	4.417*** [2.755]
Bridging2	−0.296*** [−5.699]	−0.268*** [−5.335]	−0.277*** [−5.314]	−0.295*** [−5.737]	−10.78*** [−5.142]	−5.785*** [−4.432]	−5.142*** [−3.393]	−3.847*** [−2.702]
Trust	0.0453*** [5.433]		0.0354*** [4.298]	0.0356*** [4.384]	0.0125 [0.783]		0.0176 [1.552]	0.0217** [2.044]
Trust (mean)	1.221*** [28.16]		0.0639 [0.955]	0.0877 [1.331]	0.642*** [4.649]		0.141* [1.687]	0.142* [1.862]
Employed	0.278*** [34.58]	0.232*** [20.81]	0.225*** [19.74]	0.370*** [22.04]	0.0336 [0.662]	0.168*** [7.989]	0.170*** [7.586]	0.340*** [15.20]
hh_size	−0.0272*** [−7.414]	−0.0375*** [−9.901]	−0.0445*** [−11.39]	−0.0414*** [−10.46]	−0.0326*** [−5.003]	−0.0357*** [−7.392]	−0.0432*** [−9.096]	−0.0396*** [−8.763]
Education		0.0652*** [34.67]	0.0590*** [19.79]	0.0551*** [18.76]		0.0468*** [9.462]	0.0422*** [6.745]	0.0431*** [7.505]
Town_size		0.0268*** [17.42]	0.0264*** [16.82]	0.0237*** [15.27]		0.0276*** [14.04]	0.0276*** [14.25]	0.0247*** [13.88]
Stable relationship			0.0738*** [8.522]	0.0928*** [10.34]			0.0636*** [5.808]	0.0835*** [7.788]
Prof. assoc.			0.0566*** [3.828]	0.0611*** [4.201]			0.0454** [2.481]	0.0548*** [3.298]
Sports, recr.			0.0631*** [5.936]	0.0486*** [4.626]			0.117*** [5.575]	0.0885*** [4.504]
Age		−0.000583 [−0.440]	−0.00428*** [−3.012]	0.00826*** [5.525]		−0.00449** [−2.342]	−0.00686*** [−3.621]	0.00594*** [3.145]
Age2		7.80e−05*** [5.487]	0.000113*** [7.485]	−1.58e−05 [−0.998]		0.000156*** [6.085]	0.000171*** [6.717]	2.92e−05 [1.189]
Choice and control			0.0163*** [9.685]	0.0155*** [9.404]			0.00713** [2.065]	0.00893*** [2.784]
Female				−0.0871*** [−10.47]				−0.0782*** [−7.827]
Housewife				0.176*** [8.040]				0.194*** [7.471]
Student				0.176*** [7.212]				0.180*** [6.522]
Retired				0.294*** [14.99]				0.306*** [13.24]
Children				−0.0622*** [−19.96]				−0.0602*** [−16.92]
Chief		−0.137*** [−16.79]	−0.126*** [−15.15]	−0.171*** [−18.58]		−0.159*** [−13.96]	−0.145*** [−12.75]	−0.181*** [−16.70]
Chief_works		0.212*** [17.37]	0.203*** [16.39]	0.237*** [18.43]		0.214*** [13.77]	0.207*** [13.68]	0.238*** [16.44]
Constant	0.828*** [23.70]	0.287*** [6.307]	0.161*** [2.746]	−0.0938 [−1.564]	−1.970*** [−4.149]	−0.995*** [−3.002]	−0.865** [−2.294]	−0.790** [−2.199]
Observations	22,054	21,969	20,900	20,696	21,817	21,720	20,690	20,490
R-squared	0.240	0.289	0.296	0.323	−1.306	−0.140	−0.029	0.149
Adjusted R-squared	0.238	0.288	0.294	0.322	−1.309	−0.142	−0.0311	0.147
Endogeneity test					75.93	28.64	15.02	7.794
Endogeneity p					0	8.73e−08	0.000106	0.00524
Sargan Chi-sq					0.172	0.234	1.994	0.838
Sargan p					0.678	0.629	0.158	0.360
Anderson−Rubin F					41.07	16.72	10.17	5.270
Anderson−Rubin p					0	5.56e−08	3.83e−05	0.00515
Underidentification Chi-sq					37.37	47.49	32.50	30.34
Underidentification p					7.67e−09	0	8.77e−08	2.58e−07

“Prof. assoc.”: respondent belongs to professional associations; “sports, recr.”: respondent belongs to sports or recreation institutions. *t* statistics in brackets. Country dummies included but not reported

*** $$p<$$ 0.01; ** $$p<$$ 0.05; * $$p<$$ 0.1

Our results have also been tested for robustness against a few sets of control variables that are known from the literature to have a significant impact on individual income, like education, age, age squared (the Mincerian wage equation, cf. Heckman et al. 2003), size of town of residence, as well as psychological factors such as the freedom of choice and control over one’s actions and living in a stable relationship. We find that better education, being employed, living in a stable relationship, residing in a bigger town or city, membership in professional organizations and sports and recreation institutions, and having the freedom of choice and control, go together with higher income. The relation between age and income, typically hypothesized to be inverted-U shaped, appears ambiguously signed and sometimes statistically insignificant due to the inclusion of a wide range of other control variables. Both the number of children (*children*) and adult persons in the household (*hh_size*) exhibit a negative association with equivalent income of the household. The latter outcome can be interpreted that the data perhaps favor a somewhat less sharp discounting of income by the number of household members in the computation of equivalent income than the one we have used in the current analysis. On average, women earn less than men.

As far as further control variables included in regressions (4) and (8) are considered, we obtain a few puzzling results. Namely, it turns out that controlling for a range of other individual characteristics, housewife, student, and retired status goes together with higher earnings.

Finally, we also control if the respondent declares to be the chief wage earner of the household, and whether the chief wage earner is employed (which has a differential impact from the respondent’s employment status only if the respondent is not the chief wage earner). As expected, the latter variable is robustly positively associated with individuals’ earnings. Surprisingly, however, chief wage earners tend to have *lower* earnings than non-chief wage earners. This last result is a consequence of the fact that already in the raw data, household incomes and equivalent incomes are (according to $t$ tests) actually statistically significantly lower for chief wage earners (predominantly men, often living alone). Earnings *per person in the household* are significantly higher if the respondent is a chief wage earner, though, because on average, chief wage earners tend to live in smaller households.

## Robustness Checks

The purpose of the current section is to provide additional extensions and robustness checks for our main set of results. It turns out that our main result is robust to: (a) including an extended set of countries (which is possible thanks to eliminating control variables with most missing values), (b) estimating the regression models with an ordered logit estimator, (c) using different definitions of individuals’ earnings as the explained variable, and (d) restricting the sample to chief wage earners. We also address the question of possible interaction effects between bridging social capital, social trust, and employment status (Growiec and Growiec 2014b).

### Larger Set of Countries

The key problem with data availability, limiting the sample size in our main study from 68 to 31 countries,^15^ concerns household size variables (i.e., number of persons in the household aged 18+, total number of persons in the household) as well as the “stable relationship” dummy. The size of the considered sample can be substantially expanded only if these important variables are dropped from the regressions. Moreover, unavailability of household size variables makes it also impossible to construct equivalent income and per capita income measures. As our explained variable, we can only use the original WVS scale of household earnings or its logarithm.

**Table 3:** Explaining individual earnings in an extended set of countries

Variables	(1)	(2)	(3)	(4)	(5)	(6)	(7)	(8)
	inc_ln	inc_ln	inc_ln	inc_ln	inc_ln	inc_ln	inc_ln	inc_ln
	OLS	OLS	OLS	OLS	IV	IV	IV	IV
Bridging	0.509*** [14.77]	0.349*** [9.430]	0.360*** [8.736]	0.363*** [8.991]	9.074*** [7.741]	8.472*** [6.319]	7.789*** [5.518]	6.599*** [4.815]
Bridging2	−0.289*** [−9.110]	−0.209*** [−6.205]	−0.281*** [−7.383]	−0.303*** [−8.077]	−7.907*** [−7.589]	−7.366*** [−6.238]	−6.825*** [−5.491]	−5.804*** [−4.812]
Employed	0.214*** [44.09]	0.158*** [23.29]	0.177*** [23.17]	0.293*** [26.96]	0.0831*** [3.392]	0.0669*** [3.049]	0.0720*** [3.163]	0.213*** [9.772]
Trust	0.0405*** [7.604]	0.0452*** [8.241]	0.0421*** [6.839]	0.0468*** [7.671]	0.0242*** [2.762]	0.0208** [2.147]	0.0205** [2.193]	0.0247*** [2.800]
Trust (mean)	1.197*** [34.84]	0.161*** [4.287]	0.0971** [2.256]	0.101** [2.362]	0.954*** [12.97]	0.0853 [1.410]	0.0635 [1.082]	0.0789 [1.422]
Education		0.0676*** [52.38]	0.0644*** [40.30]	0.0680*** [42.57]		0.0504*** [13.10]	0.0479*** [12.63]	0.0527*** [15.57]
Town_size		0.0225*** [20.91]	0.0178*** [15.06]	0.0199*** [16.91]		0.0220*** [12.55]	0.0211*** [12.23]	0.0216*** [13.24]
Age		0.00941*** [10.59]	0.00876*** [8.971]	0.0112*** [10.61]		0.00676*** [4.844]	0.00794*** [5.948]	0.00872*** [5.817]
Age2		−7.53e−05*** [−7.787]	−6.51e−05*** [−6.172]	−0.000111*** [−9.924]		−3.80e−06 [−0.216]	−1.93e−05 [−1.157]	−5.44e−05*** [−2.780]
Prof. assoc.			0.0962*** [9.523]	0.0972*** [9.651]			0.0779*** [5.511]	0.0814*** [6.191]
Sports, recr.			0.0939*** [12.07]	0.0768*** [9.848]			0.141*** [10.22]	0.121*** [8.638]
Choice and control			0.0189*** [15.98]				0.0119*** [5.705]	0.0121*** [6.045]
Female				−0.143*** [−23.86]				−0.117*** [−12.33]
Housewife				0.147*** [11.10]				0.189*** [9.309]
Student				0.159*** [10.45]				0.145*** [7.374]
Retired				0.221*** [16.16]				0.209*** [11.85]
Children				0.0104*** [5.091]				0.0151*** [5.437]
Chief		−0.197*** [−35.69]	−0.214*** [−35.53]	−0.276*** [−42.73]		−0.242*** [−24.56]	−0.239*** [−25.45]	−0.283*** [−33.91]
Chief_works		0.252*** [32.70]	0.261*** [30.53]	0.270*** [30.55]		0.275*** [22.54]	0.272*** [23.03]	0.270*** [23.86]
Constant	1.035*** [46.32]	0.625*** [21.90]	0.551*** [17.65]	0.578*** [18.19]	−0.713*** [−3.062]	−1.062*** [−3.741]	−0.977*** [−3.335]	−0.786*** [−2.800]
Observations	62,882	47,832	39,217	39,563	52,830	39,674	38,840	38,334
R-squared	0.181	0.285	0.302	0.318	−0.576	−0.398	−0.277	−0.094
Adjusted R-squared	0.180	0.284	0.301	0.316	−0.578	−0.400	−0.279	−0.0956
Endogeneity test					104.4	72.63	50.76	33.52
Endogeneity p					0	0	0	7.05e−09
Sargan Chi-sq					28.45	1.601	1.576	0.256
Sargan p					9.62e−08	0.206	0.209	0.613
Anderson−Rubin F					86.75	41.19	29.23	18.73
Anderson−Rubin p					0	0	0	7.39e−09
Underidentification Chi-sq					107.8	73.45	61.11	55.55
Underidentification p					0	0	0	0

“Prof. assoc.”: respondent belongs to professional associations; “sports, recr.”: respondent belongs to sports or recreation institutions. *t* statistics in brackets. Country dummies included but not reported

*** $$p<$$ 0.01; ** $$p<$$ 0.05; * $$p<$$ 0.1

Since these steps increase the number of degrees of freedom only at the cost of incurring potentially severe omitted variables bias, we have decided to treat the current analysis as a robustness check. Fortunately, from Table 3 (constructed to be fully analogous to Table 2) we learn that the main finding of the current study—that bridging social capital exhibits an inverted-U shaped relationship with individuals’ earnings—is valid for an extended set of countries as well. Signs of all other explanatory variables are also preserved. The inverted-U shaped age profile of earnings regains its statistical significance. The impact of the mean level of social trust in the respondent’s reference group is significantly positive in OLS but not in IV regressions. While negatively related to log equivalent income, the number of children is positively associated with total household income. The signs of parameters for the chief wage earner, housewife, student, and retired status remain puzzling in the extended sample as well.

**Fig. 3:**
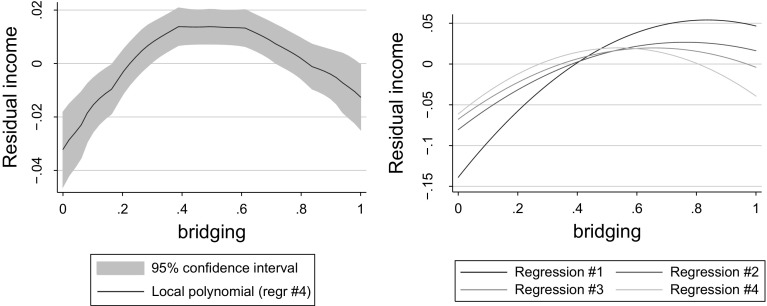
The relationship between bridging social capital and residual incomes. When based on a wider group of countries, the evidence for an inverted U is even clearer

To further assess the robustness of our inverted-U result, in Fig. 3 we have redrawn Fig. 2, using exactly the same methodology but a larger dataset and taking log incomes instead of log equivalent incomes as the explained variable. The evidence for an inverted-U relationship between bridging social capital and earnings is even clearer here: the maximum of the parabola is around 0.5 now and its right arm is visibly (and statistically significantly) declining.

### Ordered Logit Results

Another robustness check of our main set of results consists in relaxing the assumption that the WVS scale of incomes is an interval scale, in line with its “income decile” interpretation, and assuming that it is an ordinal scale instead. In such case, the appropriate empirical methodology should be based on ordered logit/probit estimates. In the following analysis, we use ordered logit. Moreover, this approach also precludes the use of equivalent incomes as the explained variable (where the income decile is divided by the square root of the number of household members).

**Table 4:** Ordered logit results

Variables	(1)	(2)	(3)	(4)	(5)	(6)
	Income	Income	Income	Income	Income	Income
	o-logit	o-logit	o-logit	o-logit	o-logit	o-logit
Bridging	2.259*** [12.56]	1.791*** [9.729]	1.699*** [9.189]	1.620*** [8.605]	1.204*** [6.195]	1.267*** [6.501]
Bridging2	−1.435*** [−8.567]	−1.244*** [−7.261]	−1.002*** [−5.852]	−0.971*** [−5.567]	−0.666*** [−3.720]	−0.965*** [−5.325]
Trust		0.160*** [5.900]		0.144*** [5.239]	0.140*** [4.978]	0.132*** [4.688]
Trust (mean)		5.025*** [34.15]		0.989*** [4.344]	1.210*** [5.239]	1.035*** [4.462]
Employed					0.781*** [19.75]	1.289*** [21.36]
hh_size	0.430*** [31.95]	0.457*** [33.25]	0.485*** [35.16]	0.485*** [34.61]	0.438*** [30.43]	0.417*** [28.44]
Education			0.257*** [39.44]	0.224*** [22.22]	0.209*** [20.22]	0.211*** [20.39]
Town_size			0.0718*** [13.76]	0.0706*** [13.30]	0.0768*** [14.16]	0.0774*** [14.24]
Age			0.0505*** [11.34]	0.0500*** [11.03]	−0.000261 [−0.0528]	0.0156*** [3.025]
Age2			−0.000425*** [−8.938]	−0.000426*** [−8.803]	0.000102* [1.943]	−0.000131** [−2.370]
Stable relationship					0.737*** [24.20]	0.657*** [21.06]
Choice and control					0.0650*** [11.20]	0.0623*** [10.72]
Female						−0.357*** [−12.24]
Housewife						0.556*** [7.150]
Student						0.528*** [5.975]
Retired						0.932*** [13.22]
Prof. assoc.						0.365*** [7.142]
Sports, recr.						0.239*** [6.576]
Chief	−0.337*** [−13.46]	−0.357*** [−14.01]	−0.437*** [−16.68]	−0.433*** [−16.25]	−0.553*** [−19.35]	−0.771*** [−23.67]
Chief_works	1.462*** [49.45]	1.369*** [45.54]	1.355*** [37.95]	1.350*** [37.23]	0.818*** [18.75]	0.890*** [19.39]
Cut1	−0.831*** [−9.212]	0.922*** [8.796]	1.269*** [8.918]	1.511*** [9.597]	1.105*** [6.584]	1.336*** [7.497]
Cut2	0.237*** [2.651]	2.017*** [19.36]	2.380*** [16.74]	2.633*** [16.76]	2.275*** [13.60]	2.523*** [14.18]
Cut3	1.125*** [12.57]	2.922*** [27.90]	3.308*** [23.16]	3.557*** [22.56]	3.235*** [19.29]	3.497*** [19.60]
Cut4	1.857*** [20.63]	3.674*** [34.79]	4.075*** [28.39]	4.323*** [27.31]	4.032*** [23.96]	4.303*** [24.03]
Cut5	2.566*** [28.32]	4.413*** [41.40]	4.831*** [33.46]	5.084*** [31.94]	4.821*** [28.52]	5.101*** [28.37]
Cut6	3.174*** [34.79]	5.051*** [46.89]	5.482*** [37.76]	5.739*** [35.86]	5.499*** [32.36]	5.786*** [32.03]
Cut7	3.857*** [41.84]	5.769*** [52.81]	6.207*** [42.44]	6.469*** [40.13]	6.251*** [36.54]	6.545*** [36.02]
Cut8	4.482*** [48.00]	6.426*** [57.93]	6.870*** [46.61]	7.136*** [43.92]	6.931*** [40.21]	7.231*** [39.53]
Cut9	5.254*** [54.96]	7.231*** [63.70]	7.684*** [51.48]	7.955*** [48.37]	7.761*** [44.52]	8.067*** [43.67]
Observations	22,848	22,145	22,273	21,589	20,939	20,938

“Prof. assoc.”: respondent belongs to professional associations; “sports, recr.”: respondent belongs to sports or recreation institutions. *z* statistics in brackets. Country dummies included but not reported

*** $$p<$$ 0.01; ** $$p<$$ 0.05; * $$p<$$ 0.1

Results of the current investigation are shown in Table 4. It turns out that the main findings of the current study are robust to the considered change in methodology. Unsurprisingly, household size is now positively related to total household earnings. The signs of all other explanatory variables are unchanged.

### Comparison Across Various Measures of Earnings

In the course of the next robustness check, we also confirm the validity of our main set of findings across various measures of earnings: (1) household earnings (WVS measure), (2) earnings per person in the household (*inc_pc*), (3) log earnings per person in the household (*inc_ln_pc*), and (4) our baseline: log equivalent earnings (*inc_ln_eq*). We maintain the assumption that earnings are measured here with an interval scale, and estimate the regression coefficients with OLS.

**Table 5:** Comparison of various income measures. OLS regressions

Variables	(1)	(2)	(3)	(4)	(5)	(6)	(7)	(8)
	Income	inc_pc	inc_ln_pc	inc_ln_eq	Income	inc_pc	inc_ln_pc	inc_ln_eq
	OLS	OLS	OLS	OLS	OLS	OLS	OLS	OLS
Bridging	1.240*** [5.702]	0.619*** [5.254]	0.470*** [7.640]	0.419*** [7.499]	1.287*** [5.952]	0.590*** [5.129]	0.460*** [7.731]	0.420*** [7.642]
Bridging2	−0.642*** [−3.190]	−0.273** [−2.505]	−0.243*** [−4.276]	−0.226*** [−4.381]	−0.969*** [-4.788]	−0.362*** [−3.362]	−0.303*** [−5.441]	−0.295*** [−5.737]
Trust	0.166*** [5.167]	0.0590*** [3.392]	0.0367*** [4.042]	0.0366*** [4.446]	0.157*** [4.910]	0.0603*** [3.544]	0.0361*** [4.107]	0.0356*** [4.384]
Trust (mean)	1.601*** [6.169]	0.675*** [4.798]	0.0971 [1.322]	0.0962 [1.443]	1.419*** [5.469]	0.702*** [5.086]	0.110 [1.537]	0.0877 [1.331]
Employed	0.872*** [19.69]	0.397*** [16.52]	0.244*** [19.48]	0.226*** [19.86]	1.353*** [20.47]	0.499*** [14.20]	0.381*** [20.98]	0.370*** [22.04]
hh_size	0.467*** [30.70]	−0.363*** [−44.05]	−0.195*** [−45.29]	−0.0436*** [−11.17]	0.443*** [28.46]	−0.348*** [−41.92]	−0.185*** [−43.11]	−0.0414*** [−10.46]
Education	0.229*** [19.77]	0.0986*** [15.69]	0.0610*** [18.58]	0.0595*** [19.98]	0.228*** [19.68]	0.0834*** [13.55]	0.0522*** [16.40]	0.0551*** [18.76]
Town_size	0.0858*** [14.03]	0.0494*** [14.92]	0.0314*** [18.17]	0.0263*** [16.72]	0.0857*** [14.03]	0.0417*** [12.84]	0.0265*** [15.75]	0.0237*** [15.27]
Stable relationship	0.761*** [22.58]	−0.272*** [−14.88]	−0.0690*** [−7.235]	0.0760*** [8.780]	0.687*** [19.44]	−0.194*** [−10.32]	−0.0161* [−1.660]	0.0928*** [10.34]
Age	0.00179 [0.324]	−0.00633** [−2.111]	−0.00841*** [−5.365]	−0.00408*** [−2.870]	0.0195*** [3.327]	0.0218*** [6.959]	0.0107*** [6.609]	0.00826*** [5.525]
Age2	9.47e−05 [1.611]	0.000245*** [7.683]	0.000197*** [11.84]	0.000111*** [7.366]	−0.000158** [−2.551]	6.84e−06 [0.207]	2.08e−05 [1.215]	−1.58e−05 [−0.998]
Choice and control	0.0705*** [10.78]	0.0263*** [7.401]	0.0161*** [8.669]	0.0163*** [9.712]	0.0683*** [10.49]	0.0249*** [7.203]	0.0152*** [8.489]	0.0155*** [9.404]
Prof. assoc.					0.417*** [7.279]	0.141*** [4.614]	0.0548*** [3.478]	0.0611*** [4.201]
Sports, recr.					0.290*** [7.011]	0.0596*** [2.712]	0.0343*** [3.018]	0.0486*** [4.626]
Female					−0.343*** [−10.48]	−0.170*** [−9.737]	−0.0778*** [−8.633]	−0.0871*** [−10.47]
Housewife					0.550*** [6.391]	0.223*** [4.872]	0.173*** [7.287]	0.176*** [8.040]
Student					0.527*** [5.486]	0.117** [2.286]	0.201*** [7.619]	0.176*** [7.212]
Retired					0.944*** [12.22]	0.264*** [6.433]	0.299*** [14.08]	0.294*** [14.99]
Children					−0.0152 [−1.240]	−0.199*** [−30.52]	−0.118*** [−34.97]	−0.0622*** [−19.96]
Chief	−0.625*** [−19.24]	−0.112*** [−6.368]	−0.0995*** [−10.82]	−0.124*** [−14.92]	−0.823*** [−22.68]	−0.190*** [−9.829]	−0.139*** [−13.89]	−0.171*** [−18.58]
Chief_works	0.845*** [17.48]	0.281*** [10.72]	0.152*** [11.08]	0.205*** [16.54]	0.913*** [18.06]	0.328*** [12.19]	0.194*** [13.95]	0.237*** [18.43]
Constant	−0.393* [−1.732]	1.168*** [9.508]	0.0875 [1.363]	0.158*** [2.705]	−0.764*** [−3.235]	0.774*** [6.158]	−0.282*** [−4.341]	−0.0938 [−1.564]
Observations	20,939	20,900	20,900	20,900	20,734	20,696	20,696	20,696
R-squared	0.400	0.301	0.328	0.294	0.413	0.338	0.378	0.323
Adjusted R-squared	0.399	0.299	0.327	0.293	0.412	0.336	0.376	0.322

“Prof. assoc.”: respondent belongs to professional associations; “sports, recr.”: respondent belongs to sports or recreation institutions. *t* statistics in brackets. Country dummies included but not reported

*** $$p<$$ 0.01; ** $$p<$$ 0.05; * $$p<$$ 0.1

As one can see in Table 5, we confirm robustness of our main result to the variety of definitions of our explained variable: the relationship between bridging social capital and earnings remains inverted-U shaped. The signs of parameters relating to other control variables are preserved as well. The only exception is the “stable relationship” dummy. While being in a stable relationship increases total household income as well as log equivalent income, it tends to decrease income per capita in the household.

It is also noteworthy to observe that while household size is statistically significantly positively related to household income (regressions (1) and (5)), it is negatively related to income per capita and equivalent income. Moreover, controlling for household size, the number of children has very similar effects: while not significantly affecting total household income, it decreases per capita and equivalent household incomes.

### Role of Chief Wage Earners

One of the biggest caveats implied by our study is the negative sign of the parameter on the chief wage earner dummy. Does this puzzling result affect the main message of the current paper? The current robustness check confirms that this is not the case.

In Table 6 we present the results of our key regression analyses—both with and without a battery of additional control variables—carried out for (i) the entire population (regressions (1) and (4)), (ii) chief wage earners only (regressions (2) and (5)), and (iii) the entire population but controlling for the chief wage earner dummy (regressions (3) and (6)). The inverted-U result is preserved across all specifications. The signs of other coefficients are also preserved.

**Table 6:** Role of chief wage earners. IV regressions

Variables	(1)	(2)	(3)	(4)	(5)	(6)
	inc_ln_eq	inc_ln_eq	inc_ln_eq	inc_ln_eq	inc_ln_eq	inc_ln_eq
	IV	IV	IV	IV	IV	IV
Bridging	5.742*** [3.500]	6.012*** [2.649]	6.207*** [3.589]	4.707*** [2.915]	4.603** [2.239]	4.417*** [2.755]
Bridging2	−4.948*** [−3.415]	−5.179** [−2.574]	−5.340*** [−3.496]	−4.109*** [−2.866]	−4.014** [−2.191]	−3.847*** [−2.702]
Trust	0.0213* [1.900]	0.0159 [1.094]	0.0181 [1.566]	0.0225** [2.078]	0.0231* [1.690]	0.0217** [2.044]
Trust (mean)	0.163* [1.959]	0.143 [1.347]	0.179** [2.106]	0.135* [1.714]	0.134 [1.397]	0.142* [1.862]
Employed	0.232*** [10.98]	0.370*** [14.68]	0.168*** [7.340]	0.393*** [18.15]	0.497*** [15.17]	0.340*** [15.20]
hh_size	−0.0207*** [−4.424]	−0.0755*** [−11.05]	−0.0418*** [−8.624]	−0.0129*** [−2.862]	−0.0650*** [−10.23]	−0.0396*** [−8.763]
Education	0.0445*** [7.254]	0.0499*** [6.395]	0.0418*** [6.597]	0.0427*** [7.239]	0.0513*** [7.210]	0.0431*** [7.505]
Town_size	0.0284*** [14.64]	0.0263*** [10.13]	0.0274*** [13.94]	0.0256*** [13.91]	0.0253*** [10.69]	0.0247*** [13.88]
Stable relationship	0.101*** [9.598]	0.153*** [11.18]	0.0666*** [6.026]	0.130*** [12.38]	0.117*** [8.710]	0.0835*** [7.788]
Age	−0.00825*** [−4.258]	−0.000634 [−0.184]	−0.00688*** [−3.545]	0.00242 [1.224]	0.00728** [2.288]	0.00594*** [3.145]
Age2	0.000151*** [5.932]	0.000112*** [2.580]	0.000173*** [6.659]	4.65e−05* [1.812]	1.39e−05 [0.349]	2.92e−05 [1.189]
Choice and control	0.00700** [2.049]	0.00658 [1.409]	0.00672* [1.918]	0.00810** [2.458]	0.00821** [1.971]	0.00893*** [2.784]
Prof. assoc.				0.0512*** [3.002]	0.0626*** [3.180]	0.0548*** [3.298]
Sports, recr.				0.101*** [5.046]	0.0959*** [4.358]	0.0885*** [4.504]
Female				−0.00201 [−0.202]	−0.128*** [−8.662]	−0.0782*** [−7.827]
Housewife				0.241*** [9.098]	0.125** [2.083]	0.194*** [7.471]
Student				0.211*** [7.479]	−0.261*** [−4.437]	0.180*** [6.522]
Retired				0.213*** [9.207]	0.250*** [7.560]	0.306*** [13.24]
Children				−0.0620*** [−17.10]	−0.0522*** [−12.05]	−0.0602*** [−16.92]
Chief			−0.143*** [−12.48]			−0.181*** [−16.70]
Chief_works			0.210*** [13.62]			0.238*** [16.44]
Constant	−0.783** [−2.166]	−1.128** [−2.400]	−0.952** [−2.493]	−0.826** [−2.285]	−0.924** [−2.142]	−0.790** [−2.199]
Observations	20,865	12,406	20,690	20,660	12,319	20,490
R-squared	−0.035	−0.020	−0.070	0.097	0.160	0.149
Adjusted R-squared	−0.0367	−0.0239	−0.0720	0.0946	0.156	0.147
Endogeneity test	15.08	8.801	16.96	9.078	5.242	7.794
Endogeneity p	0.000103	0.00301	3.82e−05	0.00259	0.0220	0.00524
Sargan Chi-sq	0.0216	3.831	0.703	0.130	2.501	0.838
Sargan p	0.883	0.0503	0.402	0.719	0.114	0.360
Anderson–Rubin F	8.714	7.962	10.25	5.515	4.787	5.270
Anderson–Rubin p	0.000165	0.000350	3.54e−05	0.00403	0.00835	0.00515
Underidentification Chi-sq	35.39	18.44	32.83	31.87	18.50	30.34
Underidentification p	2.06e−08	9.91e−05	7.44e−08	1.20e−07	9.62e−05	2.58e−07

“Prof. assoc.”: respondent belongs to professional associations; “sports, recr.”: respondent belongs to sports or recreation institutions. *t* statistics in brackets. Country dummies included but not reported

*** $$p<$$ 0.01; ** $$p<$$ 0.05; * $$p<$$ 0.1

### Interactions Between Social Capital, Trust, and Employment Status

Finally, we have also investigated if there is any interdependence between bridging social capital, social trust, and employment status in earnings regressions. Such interaction effects would be in line with the hypotheses put forward in our related theoretical study (Growiec and Growiec 2014b). In Table 7, we present a few extensions of regressions (4) and (8) from Table 2, allowing for extra interaction terms between bridging social capital, social trust, and employment status. Due to the lack of reliable instruments, we treat our interaction terms as exogenous regressors.

**Table 7:** Interactions between bridging social capital, trust, and employment status

Variables	(1)	(2)	(3)	(4)	(5)	(6)	(7)	(8)
	inc_ln_eq	inc_ln_eq	inc_ln_eq	inc_ln_eq	inc_ln_eq	inc_ln_eq	inc_ln_eq	inc_ln_eq
	OLS	OLS	OLS	OLS	IV	IV	IV	IV
Bridging	0.408*** [7.428]	0.410*** [7.471]	0.377*** [6.794]	0.379*** [6.832]	4.417*** [2.755]	4.541*** [2.783]	4.248*** [2.740]	4.368*** [2.772]
Bridging2	−0.255*** [−5.015]	−0.240*** [−4.663]	−0.304*** [−5.802]	−0.289*** [−5.454]	−3.847*** [−2.702]	−3.921*** [−2.720]	−3.564*** [−2.746]	−3.635*** [−2.767]
Bridg * trust		−0.0569* [−1.808]		−0.0613* [−1.947]		−0.119*** [−2.750]		−0.108*** [−2.703]
Bridg * empl			0.127*** [3.899]	0.129*** [3.965]			−0.224 [−1.610]	−0.230 [−1.638]
Trust	0.0367*** [4.522]	0.0666*** [3.619]	0.0365*** [4.490]	0.0686*** [3.729]	0.0217** [2.044]	0.0836*** [3.794]	0.0229** [2.228]	0.0792*** [3.742]
Trust (mean)	0.120* [1.830]	0.124* [1.890]	0.109* [1.665]	0.113* [1.727]	0.142* [1.862]	0.152* [1.959]	0.157** [1.994]	0.166** [2.082]
Employed	0.372*** [22.17]	0.372*** [22.15]	0.311*** [13.51]	0.310*** [13.44]	0.340*** [15.20]	0.338*** [14.93]	0.449*** [7.588]	0.451*** [7.558]
hh_size	−0.0411*** [−10.36]	−0.0411*** [−10.37]	−0.0409*** [−10.32]	−0.0409*** [−10.33]	−0.0396*** [−8.763]	−0.0396*** [−8.721]	−0.0400*** [−9.012]	−0.0401*** [−8.970]
Education	0.0558*** [18.96]	0.0557*** [18.94]	0.0561*** [19.09]	0.0561*** [19.07]	0.0431*** [7.505]	0.0426*** [7.285]	0.0431*** [7.326]	0.0426*** [7.127]
Town_size	0.0236*** [15.20]	0.0236*** [15.22]	0.0237*** [15.27]	0.0238*** [15.30]	0.0247*** [13.88]	0.0248*** [13.82]	0.0245*** [14.05]	0.0246*** [14.00]
Stable relationship	0.0942*** [10.48]	0.0942*** [10.49]	0.0953*** [10.60]	0.0953*** [10.61]	0.0835*** [7.788]	0.0833*** [7.718]	0.0819*** [7.424]	0.0817*** [7.352]
Age	0.00858*** [5.738]	0.00862*** [5.770]	0.00901*** [6.013]	0.00907*** [6.053]	0.00594*** [3.145]	0.00597*** [3.146]	0.00528** [2.505]	0.00528** [2.491]
Age2	−1.85e−05 [−1.168]	−1.90e−05 [−1.204]	−2.26e−05 [−1.425]	−2.33e−05 [−1.468]	2.92e−05 [1.189]	2.94e−05 [1.191]	3.43e−05 [1.266]	3.47e−05 [1.275]
Choice and control	0.0156*** [9.428]	0.0156*** [9.410]	0.0157*** [9.490]	0.0157*** [9.472]	0.00893*** [2.784]	0.00867*** [2.660]	0.00911*** [2.872]	0.00886*** [2.753]
Female	−0.0906*** [−10.91]	−0.0902*** [−10.86]	−0.0902*** [−10.87]	−0.0898*** [−10.81]	−0.0782*** [−7.827]	−0.0769*** [−7.589]	−0.0790*** [−8.095]	−0.0779*** [−7.883]
Housewife	0.176*** [8.062]	0.176*** [8.042]	0.173*** [7.907]	0.173*** [7.884]	0.194*** [7.471]	0.194*** [7.423]	0.198*** [7.383]	0.198*** [7.343]
Student	0.178*** [7.299]	0.178*** [7.295]	0.203*** [8.049]	0.203*** [8.062]	0.180*** [6.522]	0.180*** [6.479]	0.136*** [3.673]	0.134*** [3.605]
Retired	0.295*** [15.02]	0.295*** [15.02]	0.289*** [14.71]	0.289*** [14.70]	0.306*** [13.24]	0.306*** [13.17]	0.315*** [12.51]	0.315*** [12.45]
Children	−0.0622*** [−19.94]	−0.0621*** [−19.92]	−0.0620*** [−19.88]	−0.0619*** [−19.86]	−0.0602*** [−16.92]	−0.0601*** [−16.74]	−0.0607*** [−17.37]	−0.0605*** [−17.21]
Prof. assoc.					0.0548*** [3.298]	0.0554*** [3.316]	0.0587*** [3.625]	0.0593*** [3.641]
Sports, recr.					0.0885*** [4.504]	0.0906*** [4.515]	0.0899*** [4.348]	0.0920*** [4.370]
Chief	−0.171*** [−18.58]	−0.171*** [−18.55]	−0.170*** [−18.48]	−0.170*** [−18.45]	−0.181*** [−16.70]	−0.181*** [−16.61]	−0.183*** [−16.56]	−0.182*** [−16.47]
Chief_works	0.238*** [18.54]	0.238*** [18.52]	0.238*** [18.51]	0.238*** [18.49]	0.238*** [16.44]	0.238*** [16.33]	0.239*** [16.60]	0.238*** [16.49]
Constant	−0.101* [−1.693]	−0.108* [−1.797]	−0.0874 [−1.460]	−0.0940 [−1.568]	−0.790** [−2.199]	−0.832** [−2.258]	−0.768** [−2.151]	−0.809** [−2.216]
Observations	20,696	20,696	20,696	20,696	20,490	20,490	20,490	20,490
R-squared	0.322	0.322	0.323	0.323	0.149	0.139	0.166	0.156
Adjusted R-squared	0.321	0.321	0.321	0.321	0.147	0.137	0.163	0.154
Endogeneity test					7.794	8.084	7.607	7.918
Endogeneity p					0.00524	0.00447	0.00581	0.00490
Sargan Chi-sq					0.838	0.637	1.084	0.873
Sargan p					0.360	0.425	0.298	0.350
Anderson−Rubin F					5.270	5.303	5.273	5.315
Anderson−Rubin p					0.00515	0.00498	0.00514	0.00492
Underidentification Chi-sq					30.34	29.67	32.54	31.89
Underidentification p					2.58e−07	3.61e−07	8.61e−08	1.19e−07

“Prof. assoc.”: respondent belongs to professional associations; “sports, recr.”: respondent belongs to sports or recreation institutions. *t* statistics in brackets. Country dummies included but not reported

*** $$p<$$ 0.01; ** $$p<$$ 0.05; * $$p<$$ 0.1

The current extension, while not affecting the signs of coefficients estimated before, offers interesting additional findings related to the interaction terms. Statistical significance of these estimates is not fully robust to the choice of the estimation method, though, because of very strong correlations between the interaction terms and some of the interacted variables.

In particular, we find an interesting *robust* interaction effect between bridging social capital and social trust (regressions (2), (4), (6) and (8)): it turns out that the impact of bridging social capital on earnings is mediated by social trust. On the other hand, the identified interaction effect between bridging social capital and employment status is not robust.

The marginal income effects of bridging social capital, social trust, and employment status, computed according to regression (8), are as follows:

$$\begin{aligned} \frac{\partial \text {income}_i}{\partial \text {bridging}_i} = & 4.368 - 2\times 3.635 \times \text {bridging}_i - 0.108 \times \text {trust}_i -0.230 \times \text {empl}_i, \\ \frac{\partial \text {income}_i}{\partial \text {trust}_i} = & 0.0792 - 0.108 \times \text {bridging}_i, \\ \frac{\partial \text {income}_i}{\partial \text {empl}_i} = & 0.451 - 0.230 \times \text {bridging}_i, \end{aligned}$$

where for each individual, bridging social capital takes a value in the interval $[0,1]$ (the sample mean is 0.500), employment status is either zero or one (the mean is 0.639), and trust is either zero or one (the mean is 0.316). Hence, our results suggest that if one trusts strangers, additional contacts with dissimilar others are relatively less beneficial for her earnings, *ceteris paribus*; bridging social ties tend to act as a substitute for social trust. This finding aligns well with the literature which emphasizes that social trust can effectively replace information in “noisy” situations where the signals from the partner are incongruent or information on the partner is missing (Thompson 2006; Klapwijk and Lange 2009).

### Limitations of the Study

Despite our substantial effort aimed at mitigating regressor endogeneity and thus identifying exclusively the links going from social capital to earnings and not the other way round, we cannot be fully convinced that the identified links are causal. At the very core, this uncertainty is a consequence of using cross-sectional survey data which does not allow for theoretical justification of *pure* exogeneity of any of our instruments. This criticism is common to a wider body of literature, though, including the articles surveyed in Table 1.

Another potential weakness of the current study, shared with other articles based on wide survey datasets such as the WVS, lies with our empirical definition of bridging social capital as well as the choice of instruments. Our decisions on this matter, while broadly in line with the associated theoretical postulates, have been heavily constrained by data availability, somewhat compromising their reliability.

## Conclusion

The current paper has revisited the earlier studies (reviewed in Table 1) which, among other results, imply that bridging social capital generally increases individuals’ earnings. By considering the possibility of non-linear effects, our current empirical investigation fills a gap in the literature. We find that the association between bridging social capital and individual earnings is in fact *inverted-U shaped*. There is a sound theoretical explanation for such a result: the familiar positive earnings effects of bridging social ties, arising due to a range of possible resource advantages, can in fact be outweighed by negative effects—due to, e.g., time costs of social capital accumulation and maintenance—if the individual’s stock of bridging social capital is “too large”. Our empirical results are extremely robust to a wide range of manipulations in the estimated regression equations.

A methodological contribution of the current paper has been to address the endogeneity and omitted variables bias issues which are common, yet often overlooked problems in the related literature. We find these problems to be generally valid but not crucial for our principal results.

What remains to be done is to collect and use panel data to draw more precise conclusions on the causal links between social capital, trust, and economic performance of individuals and countries. Unfortunately, with WVS/EVS data we are facing an unsurmountable data availability problem in this respect.

## Footnotes

1 Table 1 reviews only studies where network operationalizations of social capital were adopted. We are particularly interested in articles acknowledging the distinction between bridging and bonding social capital. For this reason we omit the studies which take social trust as the key explanatory variable, and those which include some measures of trust as components of their social capital operationalizations (e.g., Knack and Keefer 1997; Zak and Knack 2001). In several cases, we have renamed some variables to keep them as close as possible to the current study.

2 For a theoretical, model-based elaboration of this hypothesized causal link, see Growiec and Growiec (2010, 2014b).

3 However, it must be kept in mind that as the current study is based on cross-sectional survey data, the risk of instrument invalidity can at best be assessed as low, but can never be entirely eliminated.

4 According to Bourdieu, “social capital is the sum of the resources, actual or virtual, that accrue to an individual or a group by virtue of possessing a durable network of more or less institutionalized relationships of mutual acquaintance and recognition” (Bourdieu and Wacquant 1992: 119).

5 In a working paper version of this study (Growiec and Growiec 2014c), we also included a (rather unreliable) proxy operationalization of bonding social capital via declarations of importance of family in one’s life, the content of the role of parent that one holds (following Alesina and Giuliano 2010), and the consideration of obedience as an important child quality. Crucially, adding this variable to the regressions does not affect qualitatively any of the results for bridging social capital.

6 The inverted-U relationship between bridging social capital and earnings could also be connected with the possible “transformation” of bridging ties into bonding social capital. It can be argued that, after a certain amount of time and effort has been spent in nurturing relationships with “different others”, these others are likely to become closer. Closeness could then turn into closure, leading to the formation of bonding ties that are not beneficial for earnings anymore.

7 Since most of the variables feature (at least some) missing data, the exact number of observations in each given regression specification depends on the choice of regressors and instruments. As a robustness check, we have also compared our main results against a few additional regressions where some of the relevant regressors with most missing values (such as household size) were dropped. In that case we were able to use 38–63 thousand observations from 68 countries. Our main results remain in force.

8 One could argue that friends could also be classified as bonding, not bridging social capital. From the theoretical perspective, the definition of “friends” as a group of people with whom one meets is indeed ambiguous. While—in line with our main line of distinction—they are typically non-kin (Wallace and Pichler 2007) and form dissolvable ties (Curry et al. 2013), they may also possess several important characteristics of bonding social capital (sharing similar personal characteristics, providing little resources or valuable information, offering substantial psychological support, cf. Kääriäinen and Lehtonen 2006). As the dataset doesn’t offer any information whether it is the case, we rely on its statistical characteristics, based on Cronbach’s alpha and factor analysis results, which strongly support inclusion of contacts with friends in the bridging social capital scale.

9 The approach to computing equivalent incomes by dividing total household incomes by the number of its members taken to a power $\alpha \in [0,1]$ has been first proposed by Buhmann et al. (1988). The square root assumption ($\alpha =0.5$) is likely the most popular parametrization.

10 Technically, we have used the Stata command *ivreg2*.

11 See Fan and Gijbels (1996) for a detailed description.

12 In Section 5.1 we demonstrate that for regressions based on a wider, more comprehensive sample (though missing a few relevant regressors), the current inverted-U result is even clearer.

13 Respectively: “The relationship between the amount of time invested in the formation of bridging social capital and earnings is inverse U-shaped.”; “The relationship between bridging social capital and earnings in the vicinity of the interior steady state is inverse U-shaped.”

14 For regressions including also a (highly imperfect) proxy for *bonding* social capital, see Growiec and Growiec (2014c). This latter variable appears generally negatively related to individuals’ earnings, in line with most of the literature. All other results of the current study remain essentially unaffected, though.

15 The exact list of countries is available upon request.
